# Targeted temperature management in patients with acute basilar artery occlusion after successful recanalization

**DOI:** 10.3389/fneur.2025.1553750

**Published:** 2025-08-05

**Authors:** Hong Jun Kim, Chul-Hoo Kang, Jay Chol Choi, Joong-Goo Kim

**Affiliations:** Department of Neurology, Jeju National University Hospital, Jeju National University School of Medicine, Jeju, Republic of Korea

**Keywords:** targeted temperature management, acute basilar artery occlusion, recanalization, anterior circulation stroke, acute ischemic stroke, posterior circulation stroke

## Abstract

**Background and purpose:**

Targeted temperature management (TTM) has been reported to improve outcomes in retrospective clinical studies, especially after recanalization in patients with anterior circulation stroke. However, the efficacy of TTM in managing posterior circulation stroke remains unexplored. Therefore, we investigated the clinical effects of TTM in patients with acute basilar artery occlusion who had successful revascularization.

**Methods:**

We enrolled patients with acute ischemic stroke due to basilar artery occlusion, with an initial National Institutes of Health Stroke Scale score of ≥ 10, who had successful recanalization (defined as thrombolysis in cerebral ischemia grade ≥ 2b). All patients underwent a TTM protocol targeting a core temperature of 36°C, supported by mechanical ventilation and at least 24 h of temperature management. Clinical outcomes were compared, including favorable outcomes at 3 months (defined as a modified Rankin Scale score of ≤3), mortality, length of intensive care unit (ICU) stay, and safety profiles.

**Results:**

The TTM group (*n* = 16) had a significantly lower rate of good clinical outcomes [2 (12.5%) vs. 12 (48.0%), *p* = 0.045] and a significantly longer ICU stay [11.0 (8.5–15.0) vs. 9.0 (4.0–12.0), *p* = 0.007] compared to the non-TTM group (*n* = 25). Mortality was higher in the non-TTM group, although this difference was not statistically significant. Pneumonia occurred significantly more frequently in the TTM group than in the non-TTM group (*p* = 0.004).

**Conclusion:**

TTM may not improve clinical outcomes in patients with acute basilar artery occlusion after successful recanalization.

## 1 Introduction

Despite significant advances in neuroimaging and endovascular therapy (EVT), stroke remains the second leading cause of death worldwide (1). Basilar artery occlusion (BAO) accounts for less than 1% of all strokes (2). However, it remains one of the most devastating neurological conditions associated with high risks of disability and mortality (2). Recently, the ATTENTION and BAOCHE trials showed the benefits of EVT for patients with severe symptoms of acute BAO (3, 4). Nevertheless, patients with BAO who underwent successful procedures showed higher morbidity and mortality rates compared to those who underwent successful procedures for anterior circulation stroke (5).

Targeted temperature management (TTM) has emerged as a potential therapeutic option to mitigate extensive neuronal damage and improve clinical outcomes (6–8). Although robust evidence from animal studies suggests that cooling improves outcomes after cerebral ischemia, this effect remains inadequately confirmed in patients with acute ischemic stroke (9–11). Several studies have suggested the efficacy of TTM in severe ischemic stroke involving the anterior circulation; however, its impact on posterior circulation stroke remains unexplored (9, 12).

Thus, we aim to investigate the effects of TTM (36.0°C) in patients with acute BAO presenting with severe symptoms (initial National Institutes of Health Stroke Scale [NIHSS] score of ≥10) who underwent successful revascularization of occlusive lesions after endovascular treatment. All patients received adequate sedation during the 24–144 h of cooling and rewarming in the neurological intensive care unit.

## 2 Materials and methods

### 2.1 Patient selection

Between March 2018 and February 2024, patients consecutively admitted to a single comprehensive stroke center were enrolled. The protocol for comprehensive stroke centers consists of a critical pathway for intravenous thrombolysis and EVT. Initially, all patients suspected of having acute ischemic stroke underwent computed tomography (CT) scans with three-phase CT angiography (CTA) to detect early ischemic changes and large-vessel occlusion. Within 4.5 h from symptom onset, an intravenous tissue-type plasminogen activator (t-PA) was administered with the guidelines adequately met. Based on three-phase CTA or clinical symptoms, patients suspected of having acute large-vessel occlusion were brought to the neurointerventional suite without contraindications for endovascular treatment. Endovascular treatment was performed if the onset-to-decision interval was less than 24 h, regardless of intravenous t-PA infusion. Clinical and radiologic data for these patients were reviewed. We collected information on patient demographics, vascular risk factors, imaging findings, initial vital signs, time from door to puncture, puncture to recanalization, onset to recanalization, initial NIHSS score, pre-morbid modified Rankin Scale (mRS) score, and t-PA administration. Patients were classified according to the Trial of ORG 10172 in Acute Stroke Treatment (TOAST) criteria (13). In addition, cerebral angiograms were reviewed to assess the location of occlusion and the recanalization status after EVT.

Inclusion criteria for this study were as follows: (1) acute basilar artery occlusion (NIHSS score ≥ 10) with the capability to initiate endovascular treatment within 24 h of symptom onset; (2) evidence of a small and modest early ischemic core, indicated by a posterior circulation Alberta Stroke Program Early CT Score (pc-ASPECT) greater than 6 on non-contrast CT or an overt diffusion-perfusion mismatch on initial magnetic resonance imaging (MRI); (3) confirmed successful revascularization (defined as thrombolysis in cerebral ischemia grade ≥ 2b) on digital subtraction angiography (DSA) after EVT. We excluded patients with: (1) onset to puncture time more than 24 h, (2) severe stenosis without complete occlusion, (3) other causes of stroke (e.g., arterial dissection, Moyamoya disease, or vasculitis), (4) pre-morbid mRS score greater than 2, (5) combined ischemic stroke in the anterior circulation, or (6) symptomatic intracerebral hemorrhage detected on the initial CT or MRI.

The history of all ischemic events was obtained, and the physical and neurological status of each patient was evaluated by stroke neurologists at our institution. This study was approved by the institutional review board of our hospital, and the requirement for written informed consent was waived due to the retrospective nature of the study.

### 2.2 TTM protocol

All patients received TTM using a surface cooling device (Arctic Sun, Bard Medical, Covington, GA, United States). All patients undergoing TTM were mechanically ventilated to provide airway protection, and protocolized sedation was administered (Supplementary Data 1). Cooling induction began immediately upon the patient’s arrival at the intensive care unit (ICU), with the cooling rate set to maximum until a target temperature of 36°C was reached. Temperature was recorded hourly using thermometers placed at the tip of the Foley catheter or esophageal probe. TTM was maintained for at least 24 h and could be extended to 144 h, depending on the patient’s clinical status. TTM was maintained for as long as possible during the ICU stay, but it was discontinued at the attending physician’s discretion due to factors such as refractory shivering, severe skin injury, decompressive craniectomy, end-of-life status, or guardian refusal. Rewarming was performed at a rate of 0.1–0.25°C per hour until a temperature of 37°C was achieved. Shivering was monitored using the Bedside Shivering Assessment Scale (BSAS), and a standardized protocol for shivering management was implemented (14). A detailed TTM protocol is provided in Supplementary Data 2. TTM-related clinical variables were retrospectively collected, including the mean time from recanalization to TTM initiation and the mean time from recanalization to achieving the target temperature.

### 2.3 Intervention procedure

All procedures were carried out via the percutaneous transfemoral approach under local anesthesia. Endovascular interventions were performed exclusively by two experienced interventional neurologists at our institution, who managed all cases involving large-vessel occlusions. In cases where stent placement was anticipated prior to the procedure, patients were pre-treated with a loading dose of clopidogrel (300 mg) in combination with aspirin (300–500 mg). Sedation during the procedure was determined at the discretion of the interventionists.

A 5- to 6-French long sheath (Shuttle-SL; Cook Medical, Bloomington, IN, United States) was routinely utilized for vascular access. A microcatheter with an internal diameter of 0.021 or 0.027 inches was advanced distally to the site of occlusion over a 0.014-inch steerable microwire. Angiography using the microcatheter was performed to delineate the vascular territory distal to the thrombus. Endovascular therapeutic strategies, including stent retriever and aspiration thrombectomy, were employed to achieve reperfusion in patients presenting with acute BAO. Endovascular therapy was conducted either without sedation or under conscious sedation, as deemed appropriate. The selection of thrombectomy technique—either stent retriever or direct aspiration first pass technique—was determined by the neurointerventionist. If initial attempts with stent retriever or aspiration thrombectomy were unsuccessful, alternative mechanical approaches were implemented. In cases where severe underlying atherosclerotic stenosis (>70%) with flow-limiting characteristics was identified in the basilar artery following endovascular therapy, intracranial angioplasty with or without stenting was performed, guided by the North American Symptomatic Carotid Endarterectomy Trial criteria (15). Patients who received intracranial stenting during the initial EVT, the stent placement was performed using Wingspan Stent (Stryker) without access system change. The Wingspan Stent System was deployed across the site of vessel occlusion, with or without pre- or post-balloon angioplasty. The stent length (15–20 mm) was selected to cover the entire stenotic segment. The procedure was performed in accordance with the neurointerventionist’s preferences. The diameter of the balloon was selected to be 80% of the diameter of the normal vessel immediately distal to the stenosis, and the shortest length that covered the lesion was chosen. Using an inflation device, the balloon was inflated slowly once or twice at 4–8 atm for 60–120 s. In all patients who underwent stent insertion, glycoprotein IIb/IIIa inhibitors were administered, except for those who received reactive tissue-type plasminogen activator (t-PA). Details of the interventional procedure is provided in Supplementary Table 1.

Successful recanalization was defined as achieving a modified treatment in cerebral infarction score of grade 2b or 3 (16). The recanalization time was defined as the interval between puncture and the first complete recanalization.

### 2.4 Imaging protocol

All patients underwent stroke CTA of the extra- and intracranial vessels or brain MRI, which included time-of-flight magnetic resonance angiography (MRA) of the circle of Willis and contrast-enhanced MRA. The final diagnosis of complete basilar artery occlusion was confirmed using DSA with sufficient contrast medium and prolonged imaging runs. Initial diffusion-weighted imaging (DWI) was performed immediately after the EVT. Follow-up imaging within 24–72 h after endovascular treatment comprised three-dimensional time-of-flight MRA and MRI sequences, including T2*, fluid-attenuated inversion recovery, and DWI. The basilar artery was divided into three segments according to Archer’s method (17). The proximal third of the basilar artery extends from the vertebral artery junction to the anterior inferior cerebellar artery, while the middle third spans from the origin of the anterior inferior cerebellar artery to the origin of the superior cerebellar artery. The distal third comprises the portion above the origin of the superior cerebellar artery. The occlusion location was defined as the site of the most inferior extension of the filling defect in the basilar artery. The pc-ASPECT score was determined using Tei’s method (18). Infarct volume extension, brain herniation, and the occurrence of any cerebral hemorrhage were all evaluated. Infarction volume extension was defined as an increase in lesion size when comparing the DWI performed after EVT with the follow-up image. Brain herniation was defined as the presence of transtentorial or tonsillar herniation on brain MRI. Descending transtentorial herniation is defined as the downward herniation of brain tissue, including the medial temporal lobe or the midbrain and diencephalon, through the tentorial incisura. Ascending transtentorial herniation was defined as the upward herniation of the cerebellar vermis or hemispheres through the tentorial incisura. Tonsillar herniation was considered present if one or both cerebellar tonsils herniated caudally through the foramen magnum. Any cerebral hemorrhage was defined as hemorrhagic transformation or subarachnoid hemorrhage on follow-up MRI.

### 2.5 Medical complications

All patients received standard stroke care in the ICU. Medical complications were recorded if they occurred during the admission period. These complications were classified into arrhythmia, coronary heart disease, heart failure, pneumonia, acute kidney failure, gastrointestinal bleeding, shock-requiring vasopressor therapy, deep vein thrombosis, elevated serum creatine kinase, and shivering.

### 2.6 Outcome assessment

Patient data were retrospectively retrieved from the prospective database maintained at our institute. Variables compared between the two groups (TTM vs. non-TTM) were extracted from electronic medical records. Clinical outcomes were assessed by mRS at 90 days, with scores ≤ 3 indicating functional independence and suggesting good clinical outcomes (GCO). Additional clinical and imaging outcomes included discharge NIHSS and mRS, mortality at 90 days, lengths of hospital and ICU stays, the occurrence of any cerebral hemorrhage, infarct volume extension, and brain herniation, endotracheal intubation rate, length of ventilator application, tracheostomy rate, and craniectomy rate. A neurointensivist considered patients eligible for ICU discharge if they were deemed neurologically and radiologically stable, maintained hemodynamic stability, and remained stable after being successfully liberated from major interventions such as mechanical ventilation. The detailed institutional ICU discharge criteria protocol is provided in Supplementary Data 3. Details regarding the reasons for transferring each patient from the ICU are provided in Supplementary Table 2.

### 2.7 Statistical analysis

We compared baseline characteristics, clinical status, procedural characteristics, and clinical outcomes between the TTM and non-TTM groups. Differences in baseline categorical variables were compared using the Pearson chi-squared test or Fisher’s exact test. In contrast, differences in continuous variables were compared using Student’s t-test or the Mann–Whitney *U*-test, as appropriate. Multivariate logistic regression analysis was performed to identify independent variables contributing to the outcome. Variables with a *p*-value < 0.2 in univariate analysis were included as candidate variables in the multivariate analysis and subsequently removed by backward stepwise selection. Additional analysis using forward selection confirmed the final model. A two-tailed *p*-value of < 0.05 was considered statistically significant. All statistical analyses were performed using R Statistical Software (version 2.14.0; R Foundation for Statistical Computing, Vienna, Austria).

## 3 Results

**Figure 1:**
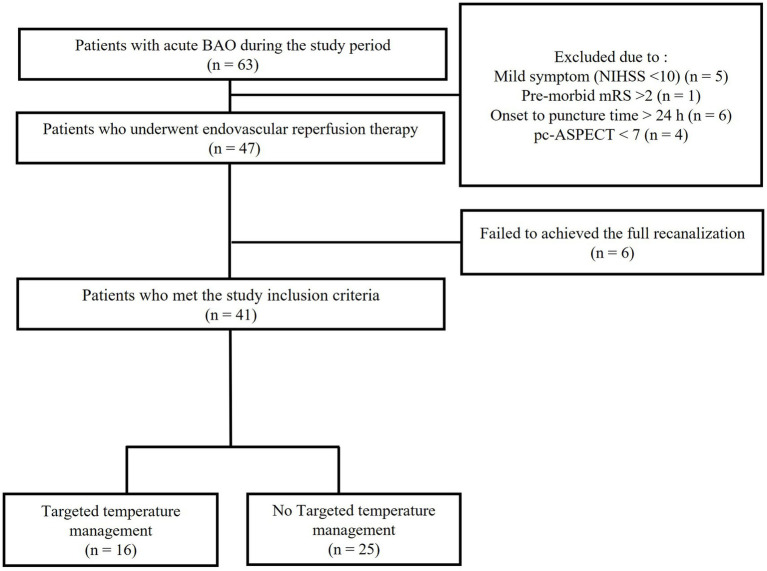
Flowchart of the patients.

The patient flowchart is shown in Figure 1. All patients had evidence of a small ischemic core, as indicated by a pc-ASPECT score greater than 6 on initial non-contrast CT or by the presence of an overt DWI–clinical mismatch. Of these 63 patients who visited our hospital for acute BAO during the study period, 16 were excluded: five due to mild symptoms (NIHSS score < 10), one due to an mRS score > 2, six due to an onset-to-treatment >24 h, and four due to a pc-ASPECT score < 7. Additionally, six EVTs performed on patients who failed to achieve full recanalization were excluded. Consequently, 41 patients with acute BAO were included in the analysis. Among these, 16 received TTM, whereas 25 were treated with standard acute stroke management. Detailed findings of hypodense lesions on the initial brain CT scans are provided in Supplementary Table 3.

There were no significant differences in baseline characteristics between the TTM and non-TTM groups without alcohol consumption (Table 1). Although the pre-stroke mRS did not differ significantly between these groups, the mean initial NIHSS [95% confidence interval (CI)] was relatively mild in the TTM group compared to the non-TTM group [20.5 (15.0–28.0) vs. 23.0 (16.0–28.0); *p* = 0.272]. The mean times from onset to recanalization [598.0 (226.0–1267.0) min vs. 390.0 (165.0–515.0) min; *p* = 0.072] and from puncture to recanalization [36.0 (20.0–63.5) min vs. 40.0 (21.0–84.0) min; *p* = 0.621] were not significantly different between the groups. No differences were noted in the proportion of patients receiving intravenous t-PA between the groups. In the TTM group, patients presented with proximal basilar artery occlusion (43.8%), mid-basilar artery occlusions (18.8%), and distal basilar artery occlusion (37.5%), whereas the respective values for the non-TTM group were 24, 16, and 60%.

**Table 1:** Baseline characteristics in patients with or without TTM.

Characteristic	Total	TTM	non-TTM	*p*
	(*N* = 41)	(*N* = 16)	(*N* = 25)	
Age	68.0 (57.0–76.0)	63.0 (58.0–68.5)	71.0 (56.0–78.0)	0.084
Sex	29 (70.7%)	13 (81.2%)	16 (64.0%)	0.405
Height	164.0 (158.0–171.0)	163.5 (158.0–171.0)	166.0 (158.0–171.0)	0.973
Weight	68.5 (55.8–75.5)	69.5 (56.0–80.0)	67.9 (55.5–74.0)	0.595
Pre-morbid mRS				0.2
0	36 (87.8%)	14 (87.5%)	22 (88.0%)	
1	5 (12.2%)	2 (12.5%)	3 (12.0%)	
Comorbidities and risk factors				
Hypertension	24 (58.5%)	7 (43.8%)	17 (68.0%)	0.225
Diabetes mellitus	7 (17.1%)	3 (18.8%)	4 (16.0%)	1
Hypercholesterolemia	5 (12.2%)	2 (12.5%)	3 (12.0%)	1
Atrial fibrillation	15 (36.6%)	6 (37.5%)	9 (36.0%)	1
Previous stroke	9 (22.0%)	5 (31.2%)	4 (16.0%)	0.445
Coronary artery disease	5 (12.2%)	1 (6.2%)	4 (16.0%)	0.659
Chronic kidney disease	3 (7.3%)	2 (12.5%)	1 (4.0%)	0.686
Smoking	12 (29.3%)	7 (43.8%)	5 (20.0%)	0.201
Alcohol	12 (29.3%)	10 (62.5%)	2 (8.0%)	0.001
Active cancer	1 (2.4%)	0 (0.0%)	1 (4.0%)	1
TOAST				0.057
Large artery disease	14 (34.1%)	9 (56.2%)	5 (20.0%)	
Cardioembolism	16 (39.0%)	4 (25.0%)	12 (48.0%)	
Undetermined source	11 (26.8%)	3 (18.8%)	8 (32.0%)	
Tissue-type plasminogen activator	13 (31.7%)	4 (25.0%)	9 (36.0%)	0.693
Initial NIHSS	24.0 (17.0–30.0)	20.5 (15.0–28.0)	23.0 (16.0–28.0)	0.272
Glasgow coma scale	9.0 (6.0–11.0)	9.0 (6.0–11.0)	8.0 (7.0–11.0)	0.697
EVT profiles				
Door to puncture time (min)	59.0 (47.0–90.0)	57.5 (45.5–88.0)	65.0 (51.0–90.0)	0.659
Puncture to recanalization time (min)	40.0 (20.0–66.0)	36.0 (20.0–63.5)	40.0 (21.0–84.0)	0.621
Onset to recanalization (min)	461.0 (225.0–765.0)	598.0 (226.0–1267.0)	390.0 (165.0–515.0)	0.072
Occlusion site				0.216
Proximal	13 (31.7%)	7 (43.8%)	6 (24.0%)	
Mid	7 (17.1%)	3 (18.8%)	4 (16.0%)	
Distal	21 (50.2%)	6 (37.5%)	15 (60.0%)	
Initial vital signs				
Systolic blood pressure	161.0 (128.0–171.0)	155.5 (121.5–176.5)	161.0 (136.0–169.0)	0.925
Diastolic blood pressure	84.0 (74.0–100.0)	88.0 (74.5–107.0)	84.0 (74.0–93.0)	0.37
Heart rate	81.0 (69.0–106.0)	92.0 (69.0–121.0)	81.0 (70.0–99.0)	0.592
Body temperature	36.6 (36.0–36.9)	36.7 (36.0–37.2)	36.4 (36.0–36.8)	0.348

TTM, targeted temperature management; mRS, modified Rankin Scale; TOAST, Trial of ORG 10172 in Acute Stroke Treatment; NIHSS, National Institutes of Health Stroke Scale; EVT, endovascular therapy.

Medical complications during ICU admission were analyzed between the TTM and non-TTM groups (Table 2). Pneumonia occurred significantly more frequently in the TTM group than in the non-TTM group (*p* = 0.004). Additionally, acute kidney injury and heart failure with pulmonary edema were statistically more common in the TTM group than in the non-TTM group (*p* = 0.036 vs. *p* = 0.019). The mean duration of TTM was 2059.5 (1802.0–4152.5) min. The mean time from recanalization to TTM initiation was 250.9 (65–146.8) min, and the mean time from recanalization to achieving the target temperature was 476.5 (185.4–767.6) min. Clinical variables related to TTM during ICU care, including TTM duration, electrolyte imbalances, shivering assessment, and shivering control, are presented in Supplementary Table 4. At discharge, the mean NIHSS was higher in the TTM group compared to the non-TTM group [18.5 (12.5–27.0) vs. 7.0 (3.0–27.0); *p* = 0.154]. However, this difference was not statistically significant (Table 3). The percentage of patients with an mRS score of 0–3 at 90 days [2 (12.5%) vs. 12 (48.0%); *p* = 0.045] was significantly higher in the non-TTM group than in the TTM group. Additionally, the length of ICU stay [11.0 (8.5–15.0) vs. 5.0 (4.0–11.0); *p* = 0.007] and the duration of ventilator use [9.0 (6.5–10.5) vs. 4.0 (3.0–7.0); *p* = 0.002] were significantly longer in the TTM group than in the non-TTM group. No significant differences were observed in infarct volume extension on follow-up imaging, herniation, tracheostomy, or posterior fossa craniectomy. The rate of hemorrhage on follow-up imaging was not significantly different between groups.

**Table 2:** Medical complications in patients with or without TTM.

Complication	Total	TTM	non-TTM	*p*
	(*N* = 41)	(*N* = 16)	(*N* = 25)	
Arrhythmia	6 (14.6%)	4 (25.0%)	2 (8.0%)	0.294
Pneumonia	18 (43.9%)	12 (75.0%)	6 (24.0%)	0.004
Acute kidney injury	4 (9.8%)	4 (25.0%)	0 (0.0%)	0.036
Gastrointestinal bleeding	0 (0.0%)	0 (0.0%)	0 (0.0%)	
Heart failure with pulmonary edema	7 (17.1%)	6 (37.5%)	1 (4.0%)	0.019
hyperCKemia	0 (0.0%)	0 (0.0%)	0 (0.0%)	
Deep vein thrombosis with/without pulmonary embolism	6 (14.6%)	3 (18.8%)	3 (12.0%)	0.886
Coronary heart disease	0 (0.0%)	0 (0.0%)	0 (0.0%)	
Shock (requiring vasopressor therapy)	6 (14.6%)	4 (25.0%)	2 (8.0%)	0.294
Shivering	15 (36.6%)	15 (93.8%)	0 (0.0%)	< 0.001

TTM, targeted temperature management; CK, creatine kinase.

**Table 3:** Comparison of clinical outcomes between the TTM and non-TTM groups.

Outcome	Total	TTM	non-TTM	*p*
	(*N* = 41)	(*N* = 16)	(*N* = 25)	
mRS 0–3 at 90 days	14 (34.1%)	2 (12.5%)	12 (48.0%)	0.045
Discharge NIHSS	14.0 (6.0–27.0)	18.5 (12.5–27.0)	7.0 (3.0–27.0)	0.154
Discharge mRS	4.0 (3.0–5.0)	5.0 (4.0–5.0)	4.0 (3.0–5.0)	0.062
Mortality at 90 days	6 (14.6%)	1 (6.2%)	5 (20.0%)	0.446
Infarct volume extension	34 (82.9%)	13 (81.2%)	21 (84.0%)	1
Any cerebral hemorrhage	6 (14.6%)	3 (18.8%)	3 (12.0%)	0.886
Brain herniation	3 (7.3%)	2 (12.5%)	1 (4.0%)	0.686
Length of ICU stays (days)	9.0 (4.0–12.0)	11.0 (8.5–15.0)	5.0 (4.0–11.0)	0.007
Length of hospital stays (days)	14.0 (10.0–24.0)	17.0 (12.0–22.5)	13.0 (8.0–26.0)	0.349
Endotracheal intubation	25 (61.0%)	16 (100.0%)	9 (36.0%)	< 0.001
Length of ventilator application (days)	6.0 (3.0–10.0)	9.0 (6.5–10.5)	4.0 (3.0–7.0)	0.002
Tracheostomy	7 (17.1%)	5 (31.2%)	2 (8.0%)	0.132
Craniectomy	1 (2.4%)	0 (0.0%)	1 (4.0%)	1

TTM, targeted temperature management; NIHSS, National Institutes of Health Stroke Scale; mRS, modified Rankin Scale; ICU, intensive care unit.

Logistic regression analysis, using GCO as the dependent variable, revealed that receiving TTM [odds ratio (OR): 0.15; 95% CI: 0.02–0.71; *p* = 0.029] was predictive of a poor outcome.

## 4 Discussion

This retrospective study compared outcomes in patients with acute BAO who underwent EVT, specifically between those treated with TTM targeting 36°C and those without TTM. No significant differences were observed between the groups regarding favorable neurologic outcomes at hospital discharge and mortality at the 3-month follow-up. However, patients in the TTM group exhibited worse clinical outcomes 3 months after stroke onset. Additionally, patients who received TTM for acute periods had a higher incidence of adverse events and a longer ICU stay.

TTM is a recommended neuroprotective intervention for all comatose/unresponsive survivors of cardiac arrest, regardless of the initial rhythm or arrest location (19). Several studies have reported that early initiation of TTM within a few hours of ischemic stroke is associated with favorable outcomes (9). Furthermore, one study reported that applying TTM following EVT in patients with anterior circulation occlusion was associated with favorable outcomes (12). The neuroprotective mechanisms of TTM in ischemic stroke appear to function by preventing glutamate accumulation in brain metabolism and neurotransmission in ischemia models. Additionally, TTM can influence many cell death pathways to favor cell survival. Thus, TTM suppresses metabolic and cellular functions while facilitating the upregulation of factors that promote cell survival. Additionally, TTM has been shown to suppress inflammatory cytokines and mediators known to elicit inflammation following ischemic stroke (10, 20, 21). However, the routine application of TTM for malignant brain infarction remains controversial due to insufficient clinical data supporting its benefits (10).

In previous clinical trials, the extended duration of therapeutic hypothermia has been limited by feasibility concerns, and low temperatures have been associated with various complications. Cooling can increase the risk of complications, such as pneumonia, coagulopathy, and bleeding, as low body temperature impairs immune function, coagulation, and platelet function (22). Endotracheal intubation and sedation for shivering control may be beneficial during TTM (23). However, ventilator-associated complications are well-known risk factors for increased morbidity and mortality (24). These complications may also extend the duration of mechanical ventilation and the length of hospital or ICU stays (25).

In this study, the proportion of patients with a favorable outcome, defined as mRS 0–3, was lower in the TTM group compared to the non-TTM group. Medical complications, such as pneumonia, pulmonary edema, and acute kidney injury during admission, were more common in the TTM group. While previous studies applied TTM for durations ranging from 6 to 72 h, this study extended its application to 144 h (9). This decision was predicated on the unclear prognostic benefits of TTM observed in prior research and the potential for secondary brain injury to persist beyond 72 h. Although the target temperature was set at 36°C to minimize temperature-related adverse effects potentially, the prolonged duration of mechanical ventilation during TTM might have contributed to increased complications, such as pneumonia and pulmonary edema, and extended the length of hospital or ICU stay. Furthermore, as a retrospective study, selection bias is possible, wherein TTM might have been more frequently applied to patients expected to have more severe symptoms.

Despite early successful revascularization, catastrophic clinical outcomes are more common in patients with posterior circulation stroke than in those with anterior circulation large-vessel occlusion (5). The ischemic thresholds differ between the anterior and posterior circulations, and clinically significant edema formation is uncommon in the posterior circulation (26). However, as the posterior fossa is a relatively narrow and crowded space, any lesion increasing its volume can abruptly elevate the intracranial pressure, leading to fourth ventricular compression and hydrocephalus (27). Thus, we hypothesized that TTM could mitigate these detrimental effects by reducing secondary brain injury, thereby improving neurological outcomes.

One strength of this study is that it is the first to validate the effect of TTM by investigating patients with severe posterior circulation stroke who achieved successful revascularization after acute BAO. In addition, it elucidates the potential medical complications of TTM in patients with acute BAO. Although retrospective, the study provides clear recommendations for interventions. Practical cooling management was performed, reflecting actual clinical practice and thus increasing the generalizability of the findings. Initial intensive care management was conducted by a single neurointensivist in both groups.

Nevertheless, our study has some limitations. As a retrospective, non-randomized registry study, it was subject to bias and unable to establish causality. Also, it is difficult to exclude the possibility that TTM was applied to patients with more severe clinical symptoms during the study period. Secondly, the effectiveness of the TTM protocol used in this study has not been validated. The unvalidated protocol may add ambiguity to the interpretation of the results. Third, this study did not provide detailed research data on neuroimaging analysis. This may affect potential biases in the initial patient classification and outcome evaluation. Fourth, a relative delay in TTM application was noted, with a mean of 250.9 min elapsing from recanalization to TTM initiation, despite the intention to apply TTM as quickly as feasible following ICU admission. Above all, the small sample size may lead to many biases and amplify the limitations mentioned. Thus, well-designed, multicenter prospective studies are warranted to investigate these results.

In conclusion, applying TTM in patients with acute BAO who have undergone EVT may not contribute to improved patient outcomes.

## Data Availability

The original contributions presented in the study are included in the article/Supplementary material, further inquiries can be directed to the corresponding author.
