# Supplementary material for: Targeted temperature management in patients with acute basilar artery occlusion after successful recanalization
Source: Front Neurol. 2025 Aug 5;16:1553750. doi: 10.3389/fneur.2025.1553750 (PMC12365378; doi:10.3389/fneur.2025.1553750)
Supplement: Supplementary file 1 [file Table_1.docx]

Supplementary Material

# Supplementary Data

Supplementary Data 1. Details on the post-intubation analgosedation protocol

Supplementary Data 2. Details on the targeted temperature management protocol

Supplementary Data 3. Details on the intensive care unit discharge criteria

# Supplementary Figures and Tables

## Supplementary Tables

| Supplementary Table 1. Location of Hypodense Lesions on initial Brain CT | | |
| --- | --- | --- |
|  | TTM | no TTM |
|  | (N=16) | (N=25) |
| Right Cerebellum | 3 (18.8%) | 10 (40.0%) |
| Left Cerebellum | 1 ( 6.2%) | 9 (36.0%) |
| Midbrain | 1 ( 6.2%) | 2 ( 8.0%) |
| Pons | 0 (0%) | 0 (0%) |
| Medulla | 0 (0%) | 0 (0%) |
| Right thalamus | 3 (18.8%) | 5 (20.0%) |
| Left thalamus | 3 (18.8%) | 2 ( 8.0%) |
| Right occipial | 2 (12.5%) | 5 (20.0%) |
| Left occipital | 1 ( 6.2%) | 0 (0%) |
| CT = computed tomography; TTM = targeted temperature management | | |

| Supplementary table 2. Details of the reasons for each patient's transfer out from ICU. | | | |
| --- | --- | --- | --- |
| No. | TTM | Reason for transfer | Length of ICU stay (days) |
| 1 | Yes | The patient meets the criteria for ICU discharge. | 12 |
| 2 | Yes | The patient meets the criteria for ICU discharge. | 9 |
| 3 | Yes | The patient meets the criteria for ICU discharge. | 8 |
| 4 | Yes | The patient meets the criteria for ICU discharge. | 8 |
| 5 | Yes | The patient meets the criteria for ICU discharge. | 9 |
| 6 | Yes | The patient meets the criteria for ICU discharge. | 10 |
| 7 | Yes | The patient meets the criteria for ICU discharge. | 11 |
| 8 | Yes | The patient meets the criteria for ICU discharge. | 12 |
| 9 | Yes | Death | 11 |
| 10 | Yes | The patient meets the criteria for ICU discharge. | 15 |
| 11 | Yes | The patient meets the criteria for ICU discharge. | 15 |
| 12 | Yes | The patient meets the criteria for ICU discharge. | 5 |
| 13 | Yes | The patient meets the criteria for ICU discharge. | 4 |
| 14 | Yes | The patient meets the criteria for ICU discharge. | 20 |
| 15 | Yes | The patient meets the criteria for ICU discharge. | 19 |
| 16 | Yes | The patient meets the criteria for ICU discharge. | 19 |
| 17 | No | The patient meets the criteria for ICU discharge. | 2 |
| 18 | No | The patient meets the criteria for ICU discharge. | 3 |
| 19 | No | The patient meets the criteria for ICU discharge. | 9 |
| 20 | No | The patient meets the criteria for ICU discharge. | 16 |
| 21 | No | The patient meets the criteria for ICU discharge. | 4 |
| 22 | No | The patient meets the criteria for ICU discharge. | 11 |
| 23 | No | The patient meets the criteria for ICU discharge. | 18 |
| 24 | No | The patient meets the criteria for ICU discharge. | 5 |
| 25 | No | The patient meets the criteria for ICU discharge. | 4 |
| 26 | No | Death | 5 |
| 27 | No | The patient meets the criteria for ICU discharge. | 3 |
| 28 | No | The patient meets the criteria for ICU discharge. | 4 |
| 29 | No | The patient meets the criteria for ICU discharge. | 9 |
| 30 | No | The patient meets the criteria for ICU discharge. | 10 |
| 31 | No | The patient meets the criteria for ICU discharge. | 14 |
| 32 | No | The patient meets the criteria for ICU discharge. | 12 |
| 33 | No | Death | 4 |
| 34 | No | The patient meets the criteria for ICU discharge. | 4 |
| 35 | No | The patient meets the criteria for ICU discharge. | 3 |
| 36 | No | The patient meets the criteria for ICU discharge. | 8 |
| 37 | No | Death | 3 |
| 38 | No | Death | 24 |
| 39 | No | Death | 3 |
| 40 | No | The patient meets the criteria for ICU discharge. | 6 |
| 41 | No | The patient meets the criteria for ICU discharge. | 4 |
| TTM = targeted temperature management, ICU = intensive care unit | | | |

| Supplementary Table 3. TTM-related clinical variables during ICU care | |
| --- | --- |
|  | TTM |
|  | (N=16) |
| TTM duration, min | 2059.5 (1802.0–4152.5) |
| Time from recanalization to TTM initiation | 250.9 (65–146.8) |
| Time from recanalization to target temperature | 476.5 (185.4–767.6) |
| Target temperature | 36C |
| Electrolyte imbalance (hypo- or hyper) |  |
| Sodium | 6 (37.5%) |
| Pottasium | 8 (50.0%) |
| Phosphorus | 10 (62.5%) |
| Magnesium | 0 (0%) |
| Shock | 3 (18.8%) |
| Shivering | 15 (93.8%) |
| BSAS |  |
| 0 | 1 ( 6.2%) |
| 1 | 2 (12.5%) |
| 2 | 5 (31.2%) |
| 3 | 8 (50.0%) |
| Shivering management |  |
| Acetaminophen | 15 (93.8%) |
| Buspiron | 12 (75.0%) |
| Magnesium | 6 (37.5%) |
| Pethidine | 4 (25.0%) |
| Dexmedetomidine | 5 (31.2%) |
| Warming blanket | 16 (100.0%) |
| Mannitol or hypertonic saline | 10 (62.5%) |
| ICU = intensive care unit; TTM = targeted temperature management; BSAS = bedside shivering assessment scale | |

| Supplementary table 4. Details of the Interventional Procedure | | | | |
| --- | --- | --- | --- | --- |
|  | Total | TTM | non-TTM | p |
|  | (N=41) | (N=16) | (N=25) |  |
| Stent placement | 9 (22.0%) | 5 (31.2%) | 4 (16.0%) | 0.45 |
| Only balloon angioplasty | 1 (2.4%) | 1 (6.2%) | 0 (0.0%) | 1 |
| DAPT loading | 5 (12.2%) | 2 (12.5%) | 3 (12.0%) | 1 |
| Clot removal with a single pass of the thrombectomy device | 31 (75.6%) | 12 (75.0%) | 19 (76.0%) | 1 |
| TTM = targeted temperature management; DAPT = Dual Antiplatelet Therapy | | | | |
